# *TP53* mutations as drivers of chordoma progression and hallmarks of aggressive chordoma

**DOI:** 10.1186/s40478-025-02180-z

**Published:** 2025-12-19

**Authors:** Szymon Baluszek, Paulina Kober, Michał Wa̧grodzki, Jacek Kunicki, Bartosz Wojtaś, Paulina Szadkowska, Bożena Kamińska, Thibault Passeri, Tomasz Mandat, Mateusz Bujko

**Affiliations:** 1 Department of Molecular and Translational Oncology, Maria Sklodowska-Curie National Research Institute of Oncology, Warsaw, Poland; 2 Department of Cancer Pathomorphology, Maria Sklodowska-Curie National Research Institute of Oncology, Warsaw, Poland; 3 Department of Neurosurgery, Maria Sklodowska-Curie National Research Institute of Oncology, Warsaw, Poland; 4 Laboratory of Sequencing, Nencki Institute of Experimental Biology PAS, Warsaw, Poland; 5 Laboratory of Molecular Neurobiology, Nencki Institute of Experimental Biology PAS, Warsaw, Poland; 6 Department of Neurosurgery, Lariboisière Hospital, Assistance Publique - Hôpitaux de Paris, University of Paris Cité, Paris, France; 7 Department of Genetics, Institut Curie, Paris, France

**Keywords:** Chordoma, TP53, DNA sequencing, Dedifferentiated chordoma, Poorly differentiated chordoma

## Abstract

**Introduction:**

Dedifferentiated (DC) and poorly differentiated chordomas (PDC) are rare, aggressive chordomas with a significantly worse prognosis than conventional chordomas (CC). The molecular mechanisms driving them remain poorly understood.

**Methods:**

Matched primary CC and recurrent DC cryopreserved samples from one patient were analyzed with whole-exome sequencing (WES). Samples from three additional DCs and one PDC underwent targeted sequencing of cancer-related genes. Furthermore, 102 CC cases - 32 novel and 70 from literature, were analyzed. Functional and survival analysis was performed.

**Results:**

WES revealed striking genomic changes during progression from CC to DC, with the number of somatic mutations increasing from 211 in primary to 430 in the recurrent DC; recurrence acquired *TP53* and *BRCA1* deleterious mutations, along with copy-number alterations, including loss of 6q containing the *TBXT* locus. Targeted sequencing identified *TP53* mutations in 4/5 DC&PDC cases compared to 1/102 cases in combined CC cohorts (*p* = 2.7$\times$10^−5^, OR=162.9). In 3 recurrent DC samples with *TP53* variant, presence of the mutation was assessed in primary CC sample and in neither, this variant was found. Literature review revealed *TP53* mutations in 9/23 (39%) DC&PDC cases versus 5/445 (1.24%) CC cases. Survival analysis demonstrated that *TP53* mutations confer a significantly worse prognosis in DC patients (*p* = 0.03).

**Conclusion:**

*TP53* mutations are acquired during chordoma progression and are associated with an aggressive phenotype; *TP53* sequencing could serve as a prognostic and potentially predictive biomarker in aggressive chordomas.

**Supplementary Information:**

The online version contains supplementary material available at 10.1186/s40478-025-02180-z.

## Introduction

Chordomas are rare bone tumors that arise from remnants of the notochord. [18] They are most often located in the sacral spine (approximately 50%) and the skull base (approximately 30%), and less frequently along the mobile spine. The main histological subtypes of chordomas include: conventional or classic chordoma (CC), dedifferentiated chordoma (DC), and poorly differentiated chordoma (PDC). Conventional chordomas are the most common, accounting for 85-90% of cases. [18, 29] Two subtypes - dedifferentiated and poorly differentiated chordomas (DC&PDCs) confer a significantly worse prognosis and are less frequent. The frequency of DCs ranges from 2 to 8%, while frequency of PDCs, a newly described entity, is probably even lower. [29, 49]

The histological characteristics of DC include high mitotic activity, necrosis, increased nuclear atypia, and lack of brachyury expression. [18] They are invasive and aggressive with a median survival of only 20 months (median survival in CC ranges from 43 to 80 months). [17, 20] For these patients treatment options remain limited and identification of biomarkers, leading to viable therapies, is of utmost importance. Dedifferentiated chordomas develop either *de novo* or as recurrent tumors after resection and/or radiotherapy of CCs. [44] Some conventional chordomas may already exhibit areas of dedifferentiation, and dedifferentiated chordomas may contain fragments of well-differentiated tumor. [18] The mechanisms and biological determinants of chordoma progression have remained unclear to date.

Yeter et al. reported only 53 cases of PDC in a literature review from 2019. [49] These tumors are aggressive, disproportionately affect the pediatric population, and are associated with SMARCB1/INI1 loss. The median survival ranges from 46 months in the general PDC population to only 9 months in PDC cases with SMARCB1/INI1 loss. [16] These tumors are brachyury-positive but display cellular atypia, increased mitotic activity, a higher tumor to stroma ratio, and loss of the structured growth pattern. [16, 18]

In this study we describe, a patient, who twice underwent extended endoscopic endonasal-transoral chordoma resection twice in a 3-year period received a histological diagnosis of classic chordoma after the first surgery and dedifferentiated chordoma after recurrence. With cryopreserved tissue samples from both surgeries and a reference blood sample from that individual, we conducted a comprehensive genomic analysis of somatic genetic changes in both tumors. We also analyzed mutations in three other patients with DC and one with PDC, who were treated at our center within the last 20 years. The cases were compared with 32 CC samples, treated at our institution, [3] and 70 publicly available CC samples. [2] The purpose of this study was to identify recurrent genetic mutations in DC&PDC and to evaluate their potential role in tumor progression.

## Materials and methods

### Patients

This study involved four patients diagnosed with a DC and one with PDC of the skull base, whose tumors underwent DNA sequencing. The group of 32 patients with conventional chordomas, whose DNA methylation profile and RNA sequencing data have been already published [3] also underwent DNA sequencing and comprised a control group. All patients underwent surgery at the Department of Neurosurgery, Maria Skłodowska-Curie National Research Institute of Oncology in Warsaw, between 2014 and 2020 and were diagnosed, according to the WHO classification criteria. The study was approved by the local Ethics Committee of the Maria Skłodowska-Curie National Research Institute of Oncology in Warsaw, Poland. Informed consent for the use of tissue samples for scientific purposes was obtained from the patients. Furthermore, 70 publicly available cases were included to extend the control group. [2]

### Nucleic acid isolation

Genomic DNA from cryopreserved tumor tissue, whole blood sample and Formalin-Fixed Paraffin-Embedded (FFPE) tissue was isolated using AllPrep DNA/RNA/miRNA Universal Kit (Qiagen), QIAamp DNA Mini Kit (Qiagen), and RecoverAll Total Nucleic Acid Isolation (Thermo Fisher Scientific), respectively. DNA concentrations were assessed spectrophotometrically using a NanoDrop 2000 (Thermo Scientific), as well as through a fluorescence-based method with a QuantiFluor Dye kit and Quantus instrument (Promega). DNA was preserved at $-20^{\circ }$C.

### Whole-exome sequencing

DNA from a blood sample, as well as primary and recurrent tumors (CC and DC, respectively) from one patient, was subjected to whole-exome sequencing (WES). Exome enrichment was performed using the Agilent SureSelectXT Reagent Kit and Agilent SureSelect Human All ExonV6 (Cat No. G9611B). A sequencing library was constructed using the Novogene NGS DNA Library Prep Set (Cat No.PT004). Libraries were sequenced using the paired end 150 bp mode on Illumina NovaSeq X Plus instrument (Illumina, San Diego, CA). The procedures were performed by an NGS service provider, Novogene.

### Targeted DNA sequencing

FFPE-derived DNA from three tumor samples of DC, one PDC, and 32 CCs was analyzed for the coding sequence of 664 cancer-related genes using the SeqCap EZ Custom Enrichment Kit (Roche, Basel, Switzerland). A complete list of genes is presented in Supplementary Table 1 and was described in details previously. [48] Briefly, one $\mu$g of DNA, isolated from each tumor sample, was processed for library preparation, following a NimbleGen SeqCap EZ Library SR (v 4.2) user guide. DNA fragmentation was achieved using Covaris M220 Focused ultrasonicator (Covaris Inc.,Woburn, MA), DNA fragment ends were repaired, and an adenosine nucleotide was introduced at the 3’ end. Indexed adapters (SeqCap Adapter Kit A and B, Roche, Basel, Switzerland) were then ligated (Ligase Enzyme from KAPA LTP Library Prep Kit, KAPA Biosystems, Woburn, MA). The libraries were amplified for seven PCR cycles (KAPA HiFi Hotstart Ready Mix, KAPA Biosystems, Woburn, MA) and subsequently mixed equimolarly with oligonucleotide probes (NimbleGen SeqCap EZ Library SR),targeting the specified genomic regions. The pooled libraries were purified after 72 h of incubation using streptavidin beads (Dynabeads M270 Streptavidin, Life Technologies, Carlsbad, CA) and amplified for 13 PCR cycles (KAPA HiFi Hotstart Ready Mix, KAPA Biosystems, Woburn, MA). The quality of libraries was assessed using the Agilent Bioanalyzer 2100 system (Agilent Technologies, CA, USA), while quantification was performed with a Quantus Fluorometer (Promega, Madison, WI).

### Sanger sequencing

The PCR amplicons of selected regions of interest were purified using ExoStar (GE Healthcare Life Sciences, Pittsburgh, PA, USA), labeled with BigDye Terminator v.3.1 (Applied Biosystems, Foster City, CA, USA), and analyzed by capillary electrophoresis with the ABI PRISM 3300 Genetic Analyzer (Applied Biosystems, Foster City, CA, USA). PCR primer sequences are presented in Supplementary Table 2.

### Data analysis

Whole-Genome Sequencing, [2] panel sequencing, and WES datasets were aligned to the GRCh38p11 human reference genome, utilizing BWA. [23] For WGS and WES data, somatic single nucleotide variants and small insertions/deletions were called using Strelka [38] and Manta [7]. Copy number alterations were identified and analyzed using the FACETS R package. [41] and samples with low tumor purity were rejected (ten WGS samples). The data and processing scripts were deposited to the Zenodo archive. [5] For panel sequencing data, given the absence of matched normal controls, variant calling was performed using Scalpel [12] and bcftools [13]. Variant annotation was subsequently conducted with vcf2maf [10], incorporating the Variant Effect Predictor (VEP). [27] Variants were filtered at a minimum of 14x coverage and a Variant Allele Frequency (VAF) of at least 0.2, with a ratio of VAF in tumor to normal control of at least 4. For samples without a normal control, common variants were rejected, and SNPs were manually checked for benign variation. Microsatellite instability was assessed with MSIsensor-pro. [19] Further analysis of MAF files was executed using the maftools R package. [26] Downstream statistical analyses and visualizations were conducted using the R v4.2.2 programming environment; graphical representations were generated using ggplot2. [15]

First, mutations and CNVs from the WES case were compared and with other CC cases from WGS in terms of mutation burden and *pfam* protein domains [34] (

**Fig. 1:**
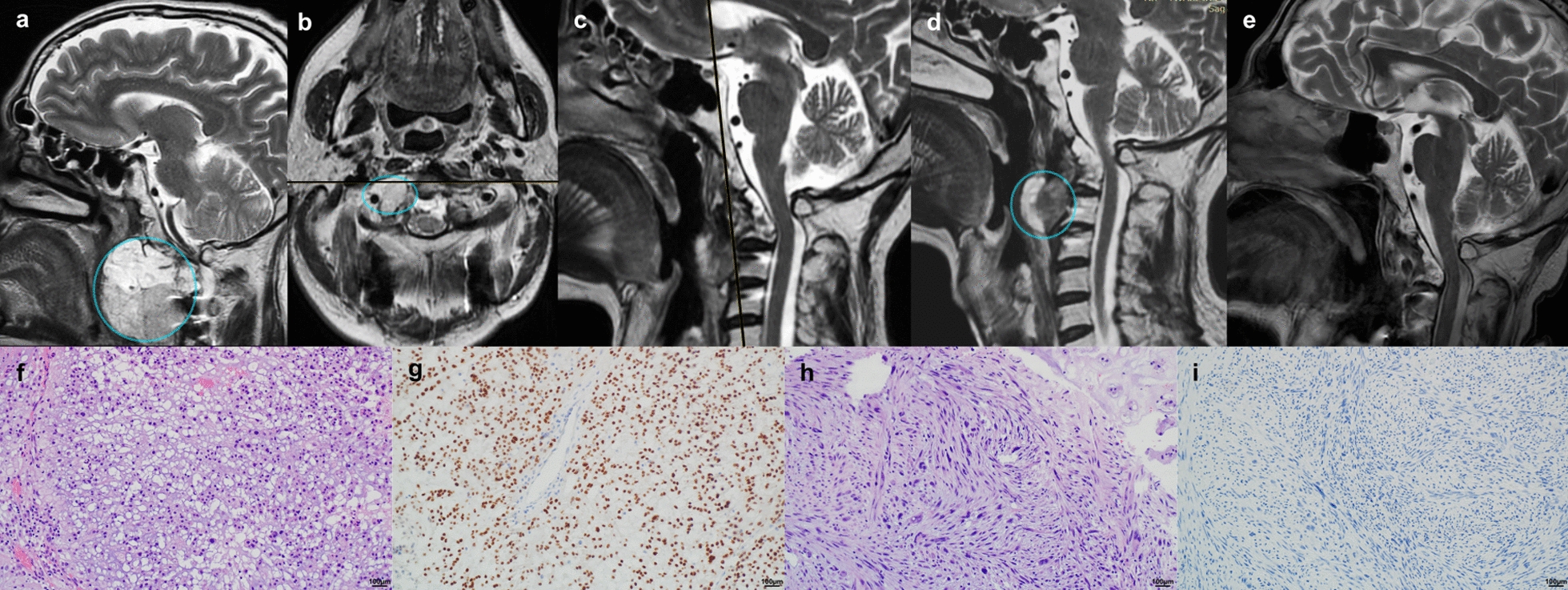
Clinical case of ZJ (primary CC and a recurrent DC); **a** MRI, sagittal section, primary tumor (CC), lesion in a cyan cycle; **b** MRI, axial section, after the subtotal first resection, remaining tumor in a cyan cycle; **c** MRI, sagittal section, after the subtotal first resection, no visible remnants on this section; **d** MRI, sagittal section, the first recurrence (DC), lesion in a cyan cycle; **e** MRI after the subtotal second resection, no visible remnants on this section; **f** H&E stained slide of the primary tumor (CC); **g** brachyury immune-stained slide of the primary tumor (CC); **h** H&E stained slide of the recurrent tumor (DC); **i** brachyury immune-stained slide of recurrent tumor (DC)

**Fig. 2:**
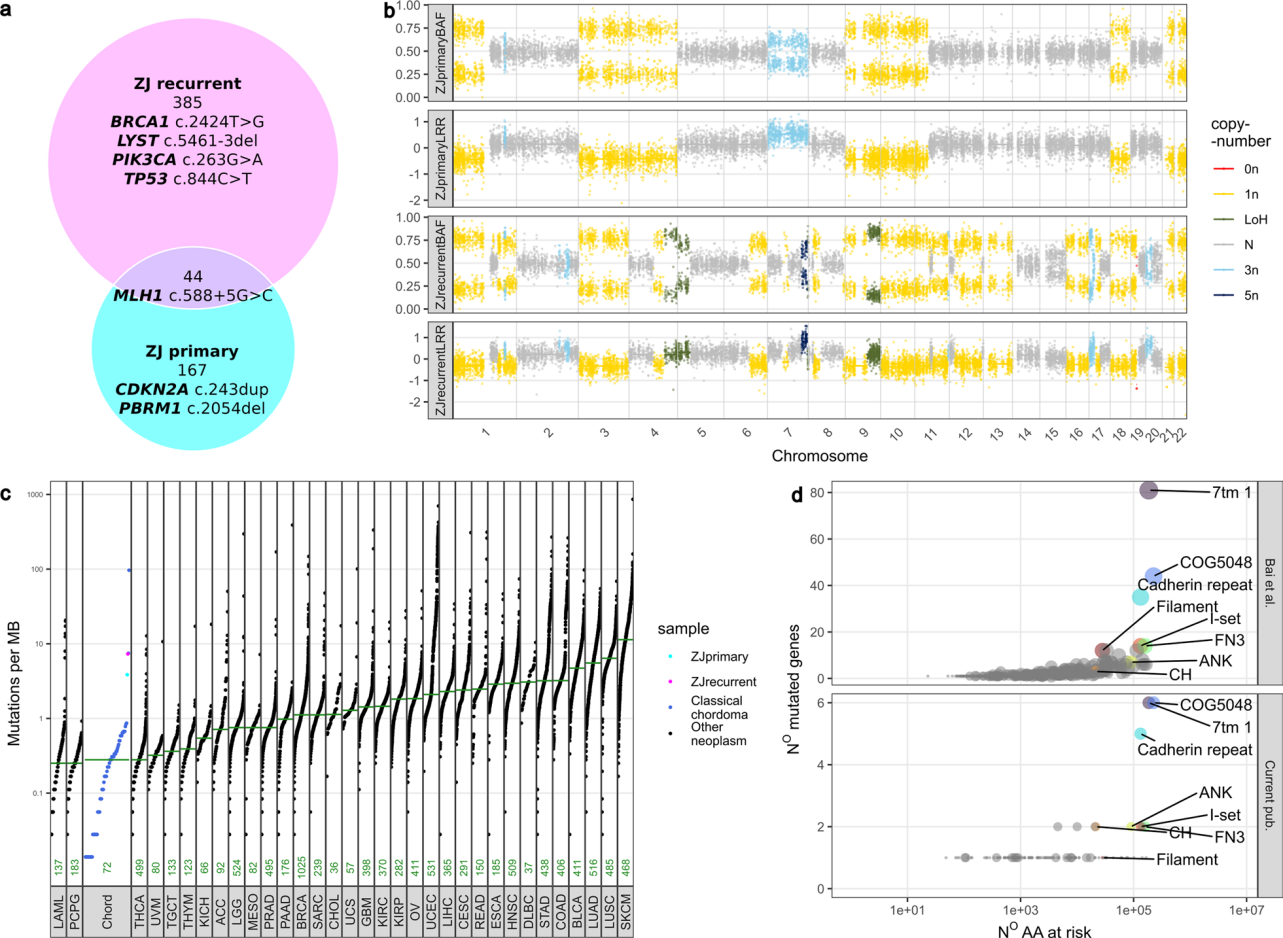
Whole-exome sequencing of ZJ (primary CC and a recurrent DC); **a** Venn diagram of mutations in primary and recurrent tumours; **b** virtual karyotype of the tumors, generated with facets; **c** Mutation burden plot, comparing primary and recurrent sample with WGS samples of CC; **d** analysis of mutations across pfam domains

Fig. 1). Next, data from panel sequencing, WES, and WGS were combined, subset to genes in the panel, and compared (exact Fisher test was applied); single base substitution profiles were extracted for each sample and compared with Catalogue Of Somatic Mutations In Cancer (COSMIC) signatures [43] utilizing cosine similarity [47] Fig. 2). Finally, *TP53* mutations reported in the literature were visualized on genomic and proteomic backgrounds; [6, 26] for TP53 visualization Mol* [40] and publicly available structure [8] were utilized. Next, the survival effect of *TP53* mutation was assessed in DC&PDC, as well as in DCs alone, utilizing both the Cox Proportional Hazards Regression Model and the Peto-Peto modification of the Gehan-Wilcoxon non-parametric test to ensure robustness. [35]

## Results

### Genetic abnormalities in progression of conventional to dedifferentiated chordoma

A 58 year-old male (ZJ) was admitted to the Neurosurgery Department with a craniocervical junction mass (Fig. 1a) diagnosed due to primary complaint of headache and upper cervical instability. He underwent biopsy and occiput-C3 instrumentation at a different institution. The primary mass was subtotally resected via an extended transnasal-transoral approach (Fig. 1b-c), and a histopathological diagnosis of CC was made. Subsequently, the patient underwent proton-beam radiotherapy. Two years later, tumor regrowth with a distinct hyperintense magnetic resonance imaging (MRI) T2 section was observed anteriorly (Fig. 1d). This lesion was subtotally resected (Fig. 1e), and this time a diagnosis of a grade 3 dedifferentiated chordoma with atypical spindle-like cells was made (see (Fig. 1f-i) for the microscopic images). Consequently, the patient received doxorubicin-cisplatin (AP3) chemotherapy. Within 12 months, progressive cervical instability, linked to tumor invasion into the cervical vertebrae, necessitated resection of the mass (reaching C6) and extending the instrumentation to Th1.

We used WES to determine somatic mutations in two tumor samples from ZJ, using peripheral blood as the germline DNA reference. A total of 211 somatic changes were found in the primary tumor (conventional chordoma), while 429 mutations were identified in the recurrent tumor (dedifferentiated chordoma). These counts include only 44 variants common to both tumors (all listed in Supplementary Table 3). See Fig. 2a for a summary. Somatic variants in genes previously reported as recurrently mutated in chordoma were found: [2, 33, 50] frameshift mutations in $CDKN2A (p.V82R)$ and $PBRM1 (p.K685R)$ along with the *LATS2* (single nucleotide deletion in 5’ UTR) in the primary tumor only. The recurrent, dedifferentiated tumor harbored a mutation in the splice site of $LYST (c.5461-3del)$, a missense mutation in $PIK3CA (p.R88Q)$, and a single nucleotide deletion in the 3’ UTR of *ANKRD17*. Importantly, the recurrent tumor also included newly acquired deleterious missense mutations in $BRCA2 (p.F808L)$ and $TP53 (p.R282W)$ at a hotspot position (rs28934574). [14]

Comparison of copy number profiles inferred from exome-seq data (based on coverage level and SNP bi-allelic frequency) showed a marked change between primary and recurrent samples. The primary tumor revealed loss of chromosomes 1p, 3, 4, 8, 9, 11p, 18, 21, and 22, as well as gain of chromosome 7. In turn, the recurrent DC did not harbor a duplication of the entire chromosome 7 but showed more focal amplification in chromosomal fragments of 7q, 17p, and 20p. This tumor also showed additional deletions compared to the primary tumor in chromosome arms 1p, 6q, 9, 11q, 13, and 19p. Of note, the 6q arm bears the region encoding for *TBXT*, a diagnostic marker found in CC, whose expression is lost in DC. Virtual karyotypes are shown in Fig. 2b. It is noteworthy that at least some of the differences in variants between primary and recurrent tumors can be explained by cytogenetic events.

Importantly, common SNVs for both samples included an *MLH1* intronic variant, mapping to 5$^{th}$ position of the splice donor site (($MLH1 c.588 + 5G > C$)), which has already been recognized as pathogenic due to deleterious effects on RNA splicing. [36] *MLH1* mutation in this patient probably results in relatively high tumor mutation burden as compared to CC samples subjected to WGS previously, as shown in Fig. 2c. The MSIsensor-pro score, reflecting frequency of microsattelite instability, reflected by polymerase *slippage*, was 5.48% (borderline) and 12.63% (high), respectively in the primary and recurrent case. [19] A comparison of affected protein *pfam* domains was also made. [34] These can reflect functional effects of the mutations. Structural function of variants, identified in ZJ and CC samples, were highly concordant. The number of mutations in a given domain was highly dependent on the number of amino acids at risk of mutation (Fig. 2d). Among the identified motifs, only cadherin domains are considered to be overrepresented in cancer. [28]

### Genetic profiles of aggressive and conventional chordomas

The hospital registry was searched for the patients diagnosed with DC&PDCs between 2008 and 2025; 4 such patients were identified:

1. A 23-year-old male (OT) was diagnosed with an incidental anterior clivus mass after a motor vehicle accident. The tumor was totally resected via an extended transnasal endoscopic surgery; a diagnosis of CC was made. The patient was observed for the next five years; at this time point, the presence of a rapidly growing mass was observed in control MRIs. The lesion was again subtotally resected, and histopathology revealed dedifferentiated chordoma with rhabdomyosarcomatous differentiation (G3); no *FOXO1* rearrangement was found. He was treated with the IVADo regimen (ifosfamide, vincristine, actinomycin D, and doxorubicin) but experienced regrowth within five months. The tumour was again near-totally removed and EP (etoposide and cisplatin) regimen was initiated but the patient died 10 months later.
2. A 64-year-old male(MM) was referred from an outside institution after biopsy of a clival chordoma. The tumor was totally resected via an extended endonasal approach, and a histopathological diagnosis of CC was confirmed. He subsequently received radiotherapy at an outside institution. After five years, he was hospitalized due to cervical instability, and MRI imaging revealed a highly cellular clival mass. He underwent cervical spine stabilization and partial tumor resection. A diagnosis of a pleomorphic dedifferentiated chordoma G3 (with spindle and epithelioid cells) was made. He died within less than three months after this surgery.
3. A 56-year-old male (DZ) underwent cervical spine stabilization and clival chordoma biopsy at an outside institution. He underwent two subsequent extended transnasal and transoral surgeries that achieved total resection of the tumor. Twice a diagnosis of dedifferentiated chordoma G2 (with spindle cells) was made. He remains active and with no significant tumor burden 97 months after the first surgery.
4. A 64-year-old (PA) female was admitted due to headache and a giant occipital tumor, diagnosed on MRI. She had primarily metastatic disease in her lungs and multiple lymph nodes. Both the occipital tumor and an inguinal lymph node were resected. Both revealed a poorly differentiated extra-axial chordoma (G3) staining for brachyury and with INI1 loss. Her clinical state was rapidly deteriorating and she died five months after the surgery.

**Fig. 3:**
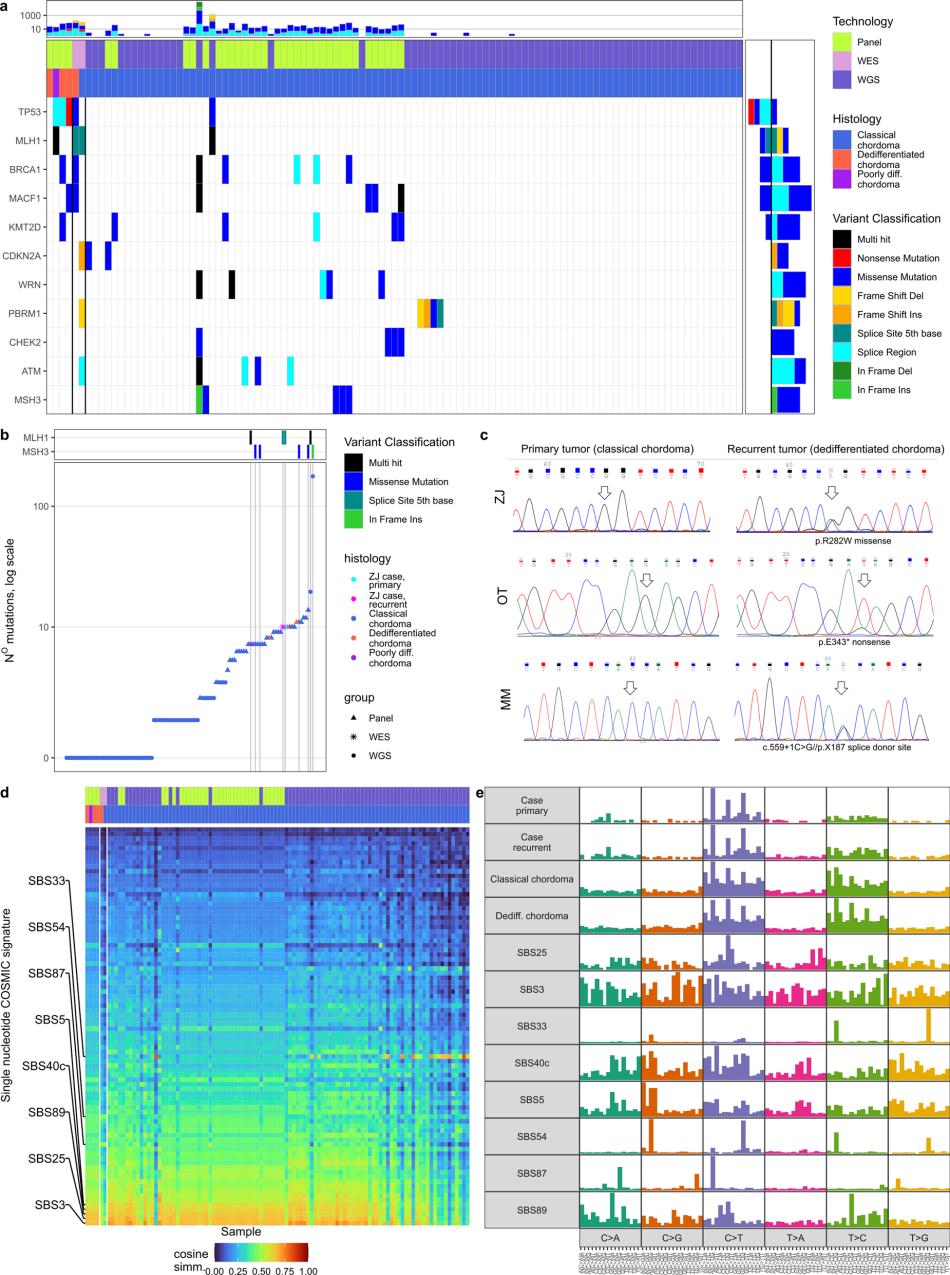
Mutations in chordoma cohorts; **a** Oncoplot of panel gene mutations, recurring in DC&PDC or CCs; **b** Mutational burden, limited to genes in the panel, mismatch-repair gene mutations are added for context; **c** Sanger sequencing of FFPE samples for patients with TP53 mutations, who suffered from CC, recurring as DC; **d** cosine similarity of the single base substitutions in samples with COSMIC signatures; **e** visualizations of mutation profiles in the samples and selected COSMIC signatures

DNA from FFPE tissue samples of these DC&PDCs was extracted and sequenced along with 32 frozen CC samples. The library was enriched for 664 cancer-related genes. Due to a lack of DNA isolated from reference germline samples, the possible somatic nature of the identified variants was inferred based on low allelic frequency in general populations, according to the adopted criteria of consecutive automatic filtration and in-person validation. These data were enhanced by including variants within the panel genes from a 70 CC WGS study by Bai et al. [2] that passed QC. The identified SNVs, considered to be somatic mutations, are listed in the associated data repository. [5] In the analyzed set of genes, we identified a median of 3.5 mutations per CC tumor (quantiles: 1.00, 1.00, 3.50, 7.25, 174.00). For the DCs, these values were 8, 10, 11, and 7 for PDC. In the case of patient ZJ, both primary and recurrent tumors had 10 mutations within the gene panel. By analyzing 664 genes in all five patients diagnosed with DC&PDC, we identified *TP53* as the most frequently mutated gene, with mutations found in 4 of 5 (80%) of the patients. All identified *TP53* SNVs are clearly pathogenic, as they include one nonsense, two splice site variants, and one missense mutation. Importantly, these five samples were compared with 32 classic chordomas (panel sequencing, current publication) and 70 classic chordomas reported in the literature (WGS, limited to the panel); this totals to 102 CC samples, bearing one *TP53* mutation. The exact Fisher test results were significant ($p = 2.7 \cdot 10^{- 5}$), $OR = 162.9$). Comparison of the recurrent mutations among panel genes is shown in Fig. 3a. No other gene was significantly enriched in DC&PDCs and only 3 were recurrent - *BRCA1*, *MACF1*, and *MLH1*.

There were relatively many mutations in the DC&PDCs samples in comparison to most CC samples. In the cases of ZJ and PA this can be attributed to the *MLH1* mutations. There existed however CC patients, who also had a higher mutational burden, in six such cases five carried a tumor with a *MSH3* mutation and one with *MLH1* mutation (Fig. 3b).

To further contextualize the role of *TP53* in CC progression to DC PCR amplification and Sanger sequencing of primary CC and secondary DC with *TP53* mutation was undertaken. All three patients with *TP53* mutation, whose DC was a recurrence (ZJ, OT, and MM), did not bear *TP53* mutation in the primary tumor (Fig. 3c).

To further explore the mutational landscape of chordoma, single base substitution (SBS) COSMIC signatures were explored, and trinucleotide matrices of all SNVs were compared by cosine similarity with existing signatures. Some signatures were highly correlated with most chordomas: $SBS3$ (associated with defective homologous recombination DNA damage repair), $SBS25$ (chemotherapy treatment), $SBS89$ (unknown etiology), $SBS40c$ (unknown etiology), and $SBS5$ (clock-like signature). Two signatures were characteristic of the ZJ case: $SBS54$ (artifact) and $SBS87$ (thiopurine chemotherapy treatment). Some other chordomas were similar to $SBS33$ of unknown etiology. The similarities are shown in Fig. 3d, and the aforementioned signatures in Fig. 3e. These results indicate a possible role of DNA repair deficiency in general chordoma oncogenesis but do not point to any striking differences in the case of ZJ or, more generally, in DC&PDCs.

### *TP53* mutations in dedifferentiated chordoma

**Fig. 4:**
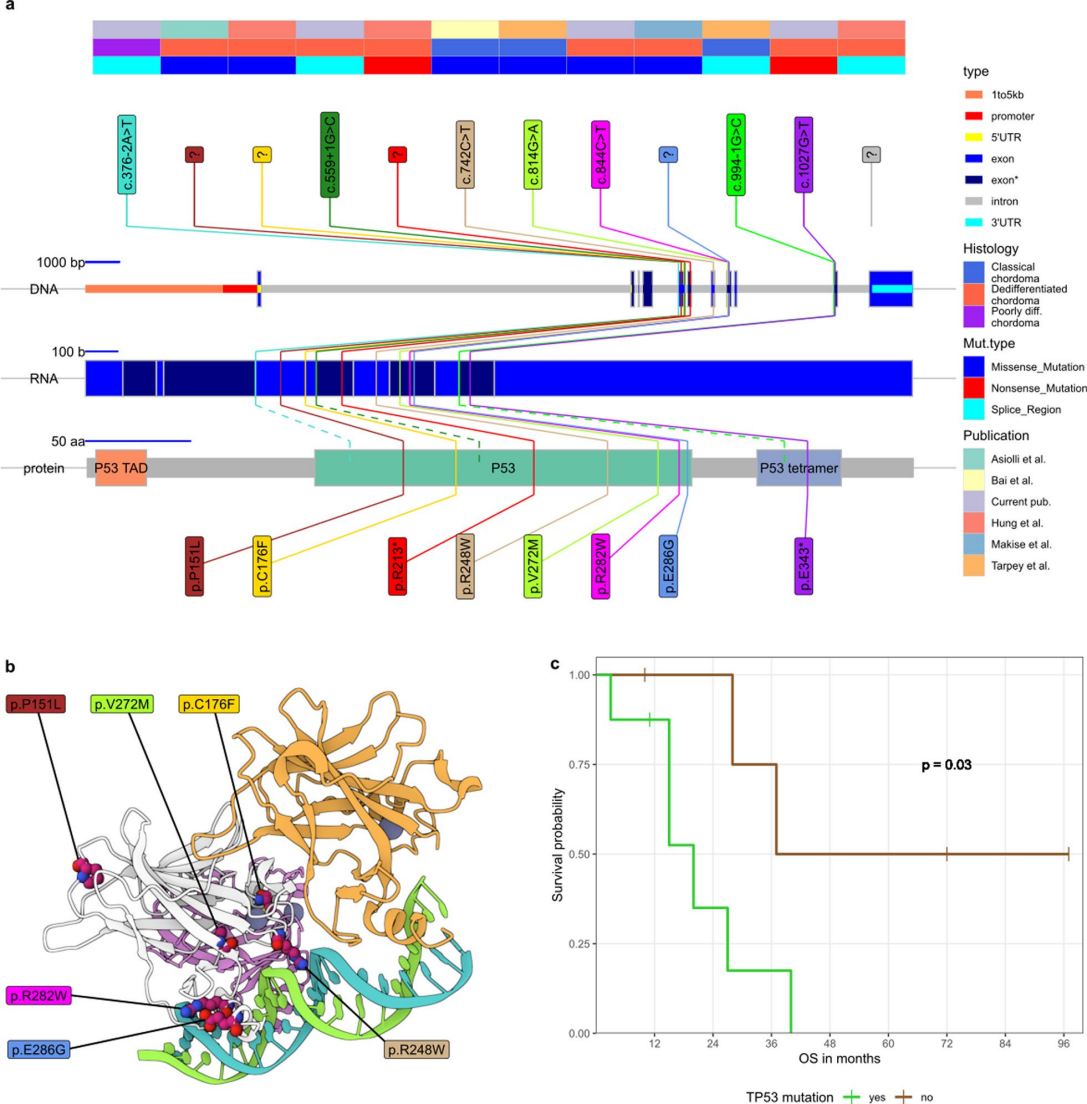
*TP53* mutations identified in all DC patients; **a** lollipop plot of mutations on DNA, mRNA, and protein level; **b** missense mutations, visualized on a TP53 binding DNA structure (PDB: TUP1); **c** Kaplan-Meier curves of overall survival for DC patients with and without *TP53* mutation, as a starting event, diagnosis of DC was used

Through a literature search, we identified studies on genomic mutations (including *TP53* mutations screening) that focused on or included DC&PDC. The reported data on immunohistochemical staining of TP53 in DC&PDC were not considered. So far, the *TP53* sequence was screened in a total of 18 DC and five PDC patients (including our series), and mutations were identified in eight (44%) and one (20%) case, respectively. These cases are listed in Table 1, and the distinct genomic and proteomic positions of all known *TP53* mutations in chordoma (including CC) are shown in Fig. 4a. Note that all reported mutations affect either the DNA-binding domain or the tetramerization domain and all the missense mutations were limited to the DNA-binding domain. The affected amino-acids were either in direct contact with DNA (Arg248, Arg282, Glu286), or possibly related to the domain structure (Val272 - hydrophobic amino acid within a domain, Cys176 - cysteine is required for disulfite bond formation, Pro151 - proline serves as a $\beta$-sheet breaker; see Fig. 4b.

**Table 1:** TP53 mutations, identified in this study course. Technologies abbreviations are as follows: panel Next-Generation Sequencing (pNGS), Whole-Exome Sequencing (WES), FoundationOne$\circledR$ CDx Test (FOT), polymerase chain reaction (PCR); Histology type abbreviations are: dedifferentiated chordoma (DC) and poorly-differentiated chordoma(PDC)

No	Variant classification	Variant	Tech- nology	Histo- logy	Follow-up [months]	Death	Key	Publication
1.	Missense Mutation	p.R282W	WES	DC	12	yes	ZJ	Current pub
2.	Nonsense Mutation	p.E343*	pNGS	DC	15	yes	OT	Current pub
3.	Splice Region	c.559+1 G>C	pNGS	DC	3	yes	MM	Current pub
4.			pNGS	DC	97	no	DZ	Current pub
5.	Splice Region	c.376-2A>T	pNGS	PDC	5	yes	PA	Current pub
6.			WES	DC	37	yes		Passeri et al. [33]
7.	Missense Mutation	p.E286G	FOT	DC	20	yes	1	Makise et al. [24]
8.			FOT	DC	72	no	2	Makise et al. [24]
9.			WGS	DC				Bai et al. [2]
10.			WGS	DC				Bai et al. [2]
11.			PCR	DC	28	yes	1	Asioli et al. [1]
12.	Missense Mutation	p.P151L	PCR	DC	40	yes	2	Asioli et al. [1]
13.	Missense Mutation	p.C176F	pNGS	DC	15	yes	B	Hung et al. [17]
14.	Nonsense Mutation	p.R213*	pNGS	DC	11	no	G	Hung et al. [17]
15.	Splice Region	unspecified	pNGS	DC	27	yes	E	Hung et al. [17]
16.			pNGS	DC	10	no	I	Hung et al. [17]
17.			WGS	PDC			PD13 455	Tarpey et al. [42]
18.			WGS	PDC			PD18 735	Tarpey et al. [42]
19.			pNGS	DC			PD22 283	Tarpey et al. [42]
20.			WES	DC			PD71 85	Tarpey et al. [42]
21.			WGS	PDC			PD18 817	Tarpey et al. [42]
22.			pNGS	PDC			PD22 308	Tarpey et al. [42]
23.			WES	DC			PD49 29	Tarpey et al. [42]

The survival data for DC patients who underwent genetic analysis were presented in articles by Hung et al. [17] (4 patients), Makise et al. [24] (2 patients), Asioli et al. [1] (2 patients), and Passeri et al. [33] (one patient). The survival information for patients first reported in this publication (four DC patients and one PDC case) was obtained from the Polish National Cancer Registry and was retrieved on January 10, 2025. Therefore, data were available for 13 DC cases bearing eight *TP53* mutations (14 and nine, respectively, including the PDC case). The overall survival was defined as the time from pathologic diagnosis of dedifferentiated chordoma (regardless of whether it was a newly diagnosed or recurrent tumor) to the time of death from any cause or to the time of the last follow-up, as adopted previously. [17] In this cohort, *TP53* mutation conferred a significantly worse prognosis (*p*=0.034 in the Peto-Peto modification of the Gehan-Wilcoxon non-parametric test); the hazard ratio in a proportional hazard model was 5.06 (95% confidence interval: 0.97$-$26.45, *p*=0.055). When the PDC case is included, the non-parametric p-value is 0.026, and the hazard ratio is 5.41 (95% confidence interval: 1.06$-$24.60, *p*=0.048). The Kaplan-Meier curves are shown in Fig. 4c.

## Discussion

Dedifferentiated and poorly differentiated chordomas are rare, aggressive histological subtypes of sarcomas, originating from the notochord. Due to the very low frequency of these tumors, little is known about their molecular pathogenesis and the mechanisms of progression from CC to DC&PDC. [18] The case of ZJ, described in our article, exemplifies chordoma progression, manifested by a change in tumor histology, and is accompanied by a marked shift in the genomic mutation profile. This patient had his tumor subtotally removed and underwent proton beam radiotherapy, but he then experienced recurrence within two years after treatment. The primary tumor was characterized by a relatively high mutational burden in comparison with classic chordomas described in the literature. [2] Of note, this tumor harbored a somatic mutation in *MLH1*, a known pathogenic variant that probably contributed to genetic instability in this patient. [36] According to the published data, mutations in mismatch repair-related genes, as well as microsatellite instability, are rare in chordomas, but in general, they are known to determine the hypermutator phenotype in human cancers. [4, 32] The secondary aggressive tumor was characterized by further accumulation of newly acquired point mutations, including pathogenic variants in genes involved in DNA repair and checkpoint mechanisms like *TP53* and *BRCA1*. These variants probably contributed to the acquisition of an aggressive phenotype in the secondary tumor and further multiple cytogenetic changes, including the loss of chromosome region 6q, which encodes *TBXT*. This chromosomal deletion probably influenced the loss of brachyury expression, a hallmark of dedifferentiated chordoma. [18]

The screening for genomic mutations in a retrospective series of additional four samples of DC was performed, and *TP53* mutations were found at a high prevalence of 80%. This observation is in stark contrast with the mutational profile of CC. We analyzed a series of 32 CCs with the same methodology, and none of the tumors harbored a pathogenic *TP53* variant. Similarly, a very low frequency of *TP53* mutations in classic chordomas was reported in the literature. Tarpey et al. [42] found 2 *TP53* mutations in a study of 104 chordomas (2/90 tumors of conventional chordoma histology); only one *TP53*-mutated CC was identified in each of the following studies: Bai *at al.* [2] (1/62 conventional chordomas), Mattox et al. [25] (1/32 chordomas of unspecified histology), and Koka et al. [22] (1/51 chordomas of unspecified histology). No *TP53* mutation were identified in studies by Wang et al. [46] (24 conventional chordomas), Passeri et al. [33] (59 calssical chordomas), nor Zhang *et. al.* [50] (95 tumors). Thus, the cumulative frequency of *TP53* mutation in conventional chordoma, calculated from the above studies, is 1.23% (5 of 444 tumors).

So far, the incidence of genomic mutations in DC&PDCs was tested in very few studies on small series of these tumors or as parts of CC cohorts. Variable methodology was used, including WGS and WES, sequencing of cancer-related gene panels, or selected *TP53* coding regions (exons 4-9). To our best knowledge, 23 DC&PDCs cases were screened for *TP53* mutations, which were identified in less than half of the DC cases, as summarized in Table 1. The particular studies differed in the frequency of mutations. Three out of four tumors were mutated in a study by Hung et al. [17], one out of two patients harbored a mutation in studies by Asioli et al. [1] and Makise et al. [24], while no *TP53* mutation was reported in DC and PDC samples by Tarpey et al. [42], Bai et al. [2], and Passeri et al. [33] We believe that methodological differences did not play a significant role in this inconsistency, as all studies but one screened the entire *TP53* sequence.

*TP53* mutations were here related to adverse prognosis in DC, as is observed in many human cancers. [37] They also play a prognostic role in some bone and soft tissue sarcomas. [30, 45] The potential of *TP53* sequencing as a prognostic factor in DC and possibly PDC, as shown by this study, should be treated cautiously and undergo further evaluation. First, the samples come from different patient populations that differ in the fraction of *TP53* mutations. There is probably heterogeneity in terms of the care provided (i.e., completeness of resection and adjuvant therapy) and possibly in histopathological evaluation (i.e. fraction of de-differentiated tissue that prompts diagnosis of DC). Moreover, other prognostic factors were shown to be important in chordoma, e.g., age at diagnosis, tumor size, primary site, metastatic status, extent of resection, and employment of adjuvant radiotherapy. Due to data heterogeneity and small sample size, it was impossible to perform a multifactor analysis that would include these factors. Importantly, other molecular factors are important in DC and PDC; for example, Makise et al. described loss of H3K27 trimethylation in half of their cohort of DCs. [24] It remains unclear whether *TP53* found in CC also confers a worse prognosis or is a risk factor for progression.

Our results indicate that *TP53* mutations are acquired during progression from CC to DC. This observation is in line with results by Hung et al., who found *TP53* mutations in three-quarters of DC cases. The authors sectioned tumor samples to separate the tissue fragments of conventional and dedifferentiated tumor. In two samples, they identified *TP53* mutations only in the dedifferentiated section. In the third tumor, the fragment of the classic chordoma had a missense *TP53* variant, but in the dedifferentiated fragment, a nonsense mutation with over 90% variant allelic frequency was found. This indicates a loss of heterozygosity at this locus. [17] Impairment of *TP53* signaling is also a feature of other dedifferentiated bone and soft tissue tumors, e.g. in dedifferentiated chondrosarcoma, a notably higher prevalence of *TP53* mutations is observed compared to classic chondrosarcoma. [11] Moreover, in dedifferentiated liposarcoma, the *TP53* pathway is downregulated by amplification and overexpression of its negative regulator *MDM2*. Consequently, a Phase III clinical trial on the use of MDM2 inhibitors (Brightline-1, NCT05218499) has been launched. [39] This illustrates that not only can *TP53* serve as a diagnostic marker, but also is potentially actionable target.

We also report on a case of metastatic PDC (PA). We found a splice region *TP53* mutation $c.376 - 2A > T$ in this patient. To our best knowledge, there have been two reports of *TP53* affected in PDC. Joldoshova et al. performed immunohistochemical staining, deduced *TP53* mutation, and confirmed it with sequencing in a recurrent sacrococcygeal PDC with INI1 loss. [21] Meanwhile, Curcio et al. reported a *TP53* relative copy-number loss along with homozygous deletion of *SMARCB1* in a case of sacral PDC. [9] Moreover, O’Halloran et al. reported some common SNPs in the *TP53* gene in their PDC cohort, but no rare *TP53* variant was identified in this publication. [31] It is important to note that *TP53* mutations can occur in PDC, and their prognostic, diagnostic, and therapeutic consequences should be further investigated in this patient population.

## Conclusion

Our findings strongly suggest that *TP53* mutations are frequently acquired during chordoma progression. These mutations appear to drive the aggressive phenotype and dedifferentiation process. This was linked to DNA repair pathway disruption and resultant genomic instability in the case, where recurrent and primary tissue was available. Consequently, *TP53* mutation status holds significant promise as a prognostic biomarker for chordomas. The *TP53* pathway can be potentially targetted, leading to much needed progress in dedifferentiated chordoma treatment.

## Supplementary Information

Supplementary material 1.

Supplementary material 2.

Supplementary material 3.

## Data Availability

Somatic mutations and copy-number segments will be freely available in Zenodo (https://doi.org/10.5281/zenodo.16751776). Sequencing data is safely stored and can be acquired for academic purposes after signing a bilateral agreement with Maria-Skłodowska Curie National Cancer Institute, Poland, Warsaw.
